# Effectiveness of interventions for children and adolescents with autism spectrum disorder in high-income vs. lower middle-income countries: An overview of systematic reviews and research papers from LMIC

**DOI:** 10.3389/fpsyt.2022.834783

**Published:** 2022-08-04

**Authors:** Maleka Pervin, Helal Uddin Ahmed, York Hagmayer

**Affiliations:** ^1^Institute of Psychology, Georg August University of Goettingen, Göttingen, Germany; ^2^Department of Psychology, University of Dhaka, Dhaka, Bangladesh; ^3^Department of Child Adolescent and Family Psychiatry, National Institute of Mental Health, Dhaka, Bangladesh

**Keywords:** interventions, autism spectrum disorder, high-income countries, lower middle-income countries, meta-review

## Abstract

**Background:**

There is a multitude of systematic reviews of interventions for children and adolescents with autism spectrum disorder (ASD). However, most reviews seem to be based on research conducted in High-Income Countries (HIC). Thus, summary findings may not directly apply to Lower Middle-Income Countries (LMIC). Therefore, we conducted a Meta-Review analyzing systematic reviews on the effectiveness of interventions for target outcomes in children and adolescents with ASD to find out whether there are differences in effectiveness between HIC and LMIC and which interventions can be considered evidence-based in LMIC.

**Methods:**

Electronic databases (PsycINFO, PubMed, Cochrane database of systematic reviews) were searched for reviews on interventions for ASD in children and adolescents from January 2011 through December 2021, which included studies not coming from HIC. Systematic reviews with qualitative and quantitative syntheses of findings were included. Two investigators independently assessed studies against predetermined inclusion/exclusion criteria and extracted relevant data including quality and evidence assessments. Evidence for different types of interventions in HIC vs. LMIC was planned to be compared, but none of the reviews assessed potential differences. Therefore, a narrative review of the studies from LMIC was conducted including an assessment of quality and evidence.

**Results:**

Thirty-five reviews fulfilled the inclusion criteria. Eleven considered findings from HIC and LMIC. Sixty-nine percent included studies with various research designs; 63% provided a qualitative synthesis of findings; 77% percent assessed the quality of studies; 43% systematically assessed the level of evidence across studies. No review compared evidence from HIC and LMIC. A review of the studies from LMIC found some promising results, but the evidence was not sufficient due to a small number of studies, sometimes poor quality, and small sample sizes.

**Conclusion:**

Systematic reviews on interventions for children and adolescents with ASD did not look for potential differences in the effectiveness of interventions in HIC and LMIC. Overall, there is very little evidence from LMIC. None of the interventions can be considered evidence-based in LMIC. Hence, additional research and mutually agreed methodological standards are needed to provide a more secure basis for evidence-based treatments in LMIC trying to establish evidence-based practices.

## Introduction

Autism Spectrum Disorder (ASD) is characterized by persistent deficits in social communication and social interaction across multiple contexts, including deficits in social reciprocity, in non-verbal communicative behaviors, and in skills required for developing, maintaining, and understanding relationships. In addition, restricted, repetitive patterns of behavior, interests, or activities have to be present for a diagnosis (1). Symptoms emerge during the first 3 years of life (2, 3). The symptoms of ASD vary in severity and may present differently in children with a mixture of cognitive abilities (4). The extreme variability of behavioral and communicative problems and coexisting conditions make it difficult for mental health professionals and non-specialists to identify ASD as early as possible (5, 6), although respective diagnostic tools exist for children as young as 18 months. The American Academy of Pediatrics (AAP), for example, recommends standardized screening for ASD at 18 and 24 months of age with ongoing developmental surveillance in primary care.

Worldwide, there is an increasing number of children, who meet the diagnostic criteria for ASD (7, 8). The estimated number of cases is 52 million worldwide, which means that 1–2% of children are affected (9–12). The prevalence rate for children was one in 44 in the U.S, based on a sample of 8-year-old children (13) and one in 100 in the UK (14). In Europe (Germany, Poland, France, Belgium, Denmark, Iceland, Sweden, Ireland), China, and North America the reported prevalence of ASD is close to 1.5%, but varies considerably between regions and populations (11, 15–22). The majority of the epidemiological studies were conducted in HIC. According to the World Bank (23), HIC are defined as countries with an average income of more than 12,353 U.S. dollars per year, upper middle-income countries (UMIC) by an average income between 4,046 and 12,535 dollars, and LMIC by an average income between 1,036 and 4,045 dollars per year. The prevalence rate in LMIC is rather uncertain due to a lack of research (24). A systematic review on the prevalence of ASD in Asia revealed that it was around 1.9/10,000 before 1980 and 14.8/10,000 from 1980 to 2008 (25). For South Asia, a systematic review estimated the prevalence as 0.09% in India, 1.07% in Sri Lanka, and 3% in Bangladesh (26).

ASD is considered an emerging public health issue by the World Health Organization (27). Still, research, public awareness, and mental health services are mostly concentrated in HIC. In these countries, large efforts have been made to bridge the gap between evidence and practice. By contrast, a large gap exists in LMIC due to a lack of public awareness, professional knowledge, and well-conducted scientific studies (28). International studies found that 75–85% of individuals with mental disorders including autism do not receive particular treatment services in LMIC (29), which prevents children from realizing a healthy life (30). Major barriers to increasing services for childhood mental disorders in these countries include financial constraints, absence of government initiatives, inadequately trained healthcare professionals, and an overcentralized health system (31–37). In addition, there might be limited knowledge about effective evidence-based treatments and a lack of competencies required for their implementation (38).

Many different types of treatments for children and adolescents with ASD have been developed and investigated [cf. (39)]. With respect to cognitive and/or behavioral interventions, it is important to delineate comprehensive treatment models and focused interventions. Comprehensive treatment models (CTM) are conceptually organized sets of practices, which address the core deficits of ASD over a lengthy period of time (e.g., 1–2 years). Multiple developmental domains (e.g., social communication, daily living skills, and repetitive behaviors) are targeted by using multiple interventions (e.g., The UCLA Young Autism Program by Lovaas (40), the TEACCH program developed by Lord and Schopler (41), the LEAP model, the Early Start Denver model). Many comprehensive programs aim at young children, which underlines the importance of an early diagnosis. By contrast, focused interventions are a set of individual instructional strategies that are designed to address a specific behavioral or developmental problem, for instance, joint attention or repetitive behaviors. Further examples are social skills training or visual support in academic instruction.

A special sub-group of treatments is psychosocial interventions delivered by non-specialists (parents or caregivers, peers, and teachers). In many LMIC, interventions for children and adolescents with ASD have to be delivered by these non-specialists due to a lack of other resources. Therefore, we considered these treatments separately, although the interventions themselves overlap with focussed interventions. In community settings, these interventions have been found to produce benefits in development, social-communication skills, daily living skills, comprehension or academic performance, behavior, or family outcomes (42, 43).

In recent years, technological devices have been used more often to deliver treatments, train, and support health care professionals as well as parents. Technology-based interventions make use of a broad range of devices such as speech-generating devices or robots, and software applications like computer-assisted instructional programs, or mobile- and tablet-based applications (44–47). Educational computer games (e.g., EmotionTrainer, FaceMaze, FaceSay, Squizzy, TeachTown) were designed for enhancing a broader set of skills, including social, emotional, as well as cognitive, and academic skills (48–53). As technological devices and software programs require substantial financial resources to acquire and maintain them, we decided to treat respective treatments as a separate sub-group to provide respective information for readers coming from LMIC.

In addition to cognitive and behaviorally oriented treatments, medical and alternative treatments have been developed and tested (47, 54). The use of medical treatments to address behavioral problems in children and youth with ASD has increased significantly since the publication of the AAP's clinical report in 2007 [cf. (55, 56)]. The U.S. Food and Drug Administration (FDA) has approved the use of some antipsychotic drugs, such as aripiprazole and risperidone, for the treatment of irritability/ aggression and repetitive behaviors in children and youth with ASD.

Complementary and alternative medicine (CAM) treatments refer to a broad set of health care practices that are not part of that country's own tradition and are not integrated into the dominant health care system (57). These encompass diets (e.g., gluten-free diet, ketogenic diet), nutritional supplements (e.g., omega 3 fatty acids, vitamins, melatonin), traditional alternative medicine (e.g., acupuncture), exercise (e.g., yoga), body therapies (e.g., massage, touch therapy). CAM treatments are frequently used to treat behavioral problems (e.g., aggression, irritability, hyperactivity). Some interventions classified as CAM were found to be ineffective, some potentially harmful (58, 59).

The research on the effectiveness of the different types of treatments looked at various outcomes, including language development, interpersonal skills, behavior, and academic achievement. Systematic reviews often summarize the findings for a specific type of treatment and/or for a specific type of outcome. Very few try to collate the evidence across all types of treatments [see (47, 54, 60), for exceptions]. Based on the findings, some treatments have been identified as evidence-based practices, that is, as treatments for which sufficient evidence is available that they are beneficial for the outcome under investigation. The latest review of the National Standards Project (NSP) and the National Professional Development Center (NPDC) identified 27 evidence-based practices (61, 62).

Research has shown that clinical features of ASD present the same in HIC and LMIC (63–65). However, the significant contextual differences between HIC and LMIC may result in very different consequences (66). HIC provide treatment facilities and comprehensive care for children and adolescents with ASD. A rather large number of mental health professionals with a specific focus on developmental disorders (psychiatrists as well as clinical psychologists) are available. The awareness of ASD is generally high. The situation in LMIC is rather different. In many aspects, it is quite the opposite. In most LMIC, there are very few trained professionals, who have expertise with respect to ASD-related interventions. In addition to insufficient training, there are financial constraints and limited resources within health care systems, which are much less elaborated than in HIC (33, 36, 37). Finally, there are substantial cultural differences and medical traditions in LMIC than in the mostly Western HIC. Therefore, interventions designed and tested in HIC may turn out to be less applicable and less effective in LMIC (43, 67–71). Hence, it is important to look for potential differences.

In the past two decades, many reviews (systematic and unsystematic) on treatments for children and adolescents with ASD and other developmental disorders have been published. Most of these come from researchers in HIC, although ~95% of individuals with ASD do not live in these countries (64, 72, 73). There are very few reviews that come from and focus on evidence from LMIC, although some studies have been conducted (see **Table 2** for on overview). Hence, there is a need for conducting a systematic review of reviews to summarize and compare the results from HIC and/or LMIC. This is the aim of the present meta-review. It provides an overview of the existing systematic reviews published from the beginning of 2011 up to the end of 2021, analyzes potential differences in findings from HIC and LMIC, summarizes the effectiveness of the different types of interventions, and describes the quality and findings of the studies coming from LMIC.

The following research questions were addressed:

- Do systematic reviews of treatments for children and adolescents with ASD consider research findings from LMIC?
- Are there differences in the effectiveness of interventions in HIC and LMIC?
- Which types of treatments can be considered evidence-based in LMIC?

## Methods

### Search strategy

A systematic review of reviews was carried out in accordance with the Preferred Reporting Items for Systematic Review and Meta-Analyses guidelines [PRISMA, (74)]. Eligible review articles were obtained by searching three electronic databases: PsycINFO, PubMed and Cochrane Database of Systematic Reviews. The research team developed a series of search terms appropriate for each database using medical subject headings (MeSH). These terms included “Review” OR “Review as literature” AND “Autism” OR “Autism spectrum disorders” AND “Evidence based practice” OR “Evidence based treatment” OR “Treatment program” OR “Interventions” AND “High- income countries” OR “Lower middle-income countries.” A manual search of the reference lists of all included reviews was conducted to identify additional reviews.

### Eligibility criteria

Criteria for inclusion were defined in advance. To be included, the review had to be systematic (i.e., a clear objective and research questions had to be specified and the methodology including a search and data extraction strategy had to be described in enough detail to be replicable). Reviews had to be published between January 2011 and December 2021 in English. The population had to be children and adolescents (up to 18 years of age) diagnosed with ASD. There were no restrictions with respect to treatment, treatment setting, or outcome. Reviews had to include studies coming not only from HIC. Following the classification of countries from the World Bank, this includes studies from LMIC and UMIC, although we were interested in LMIC. Note that we decided to be overinclusive at this point to provide a good overview on how research not coming from HIC is taken into account in systematic reviews. Reviews with a qualitative and/or quantitative synthesis of findings were included. Reviews not meeting these inclusion criteria were excluded.

### Study selection

**Figure 1:**
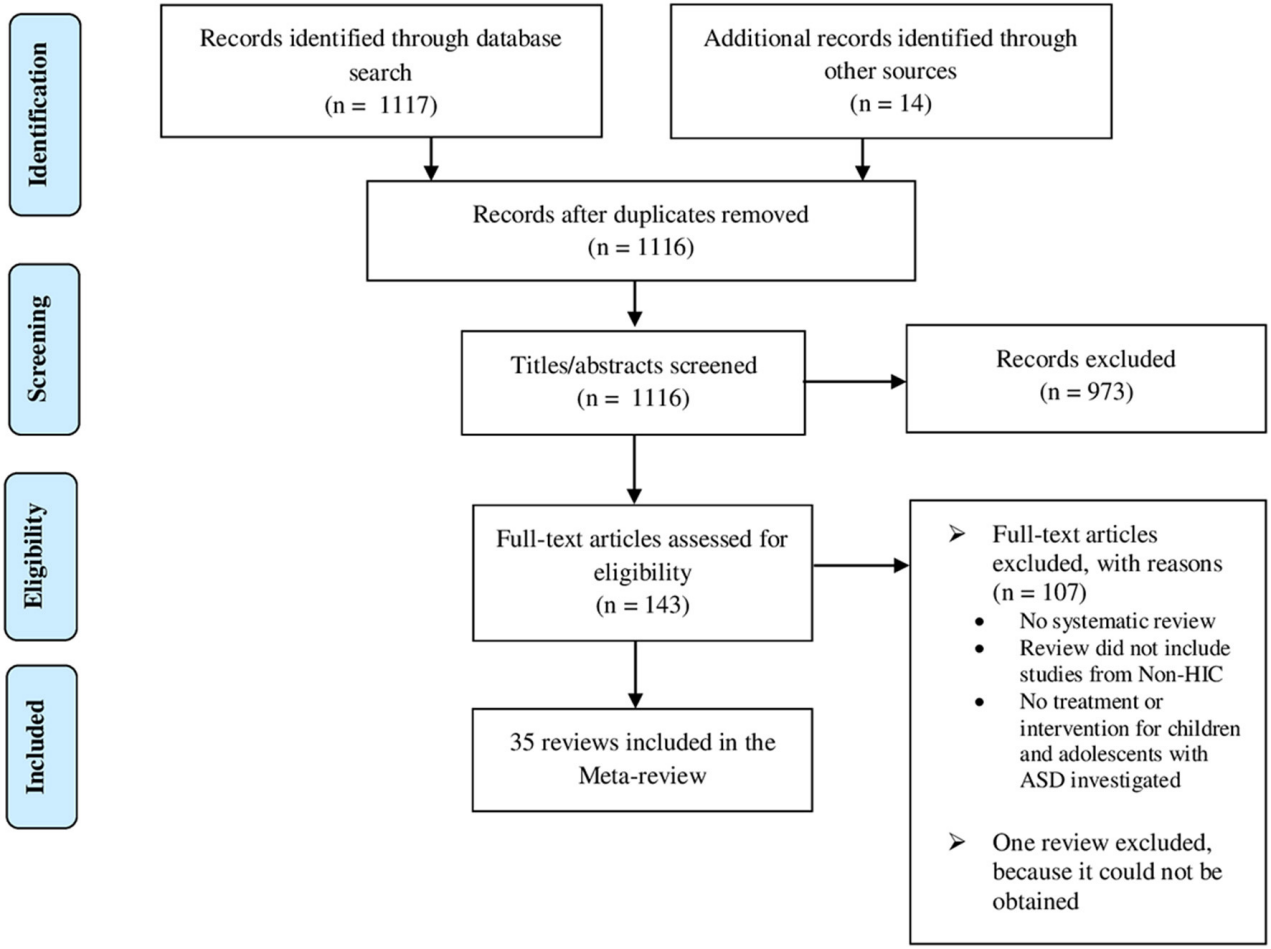
PRISMA flow diagram [modified from Moher et al. (74)].

The first author screened all review papers, initially on the basis of title and abstract to identify potentially eligible reviews. All titles and abstracts were screened independently by the third author. Following this, full articles were assessed independently by the first author and third author with respect to the inclusion criterion. Initial agreement was 92%. Disagreements were resolved through discussion. Reviews not meeting the inclusion criteria were excluded. The flow of studies is presented in the respective PRISMA diagram shown in Figure 1.

### Data extraction and management

Data were extracted from all eligible reviews and tabulated by the first reviewer using a set of data extraction forms, which were developed for the present meta-review. The following information were collected: name of the first author, year of publication, age group, countries of included studies [HIC, LMIC, or UMIC, as defined by the (23)], number of included studies, study designs, data synthesis method, quality assessment method for individual studies, method for evidence rating across studies, types of treatments, types of outcomes, evidence for each type of treatment according to the authors of the review. Treatments and outcomes were classified following the categories proposed by the National Autism Center (61, 62, 75). To provide a better overview, we organized treatments into the seven major groups outlined in the introduction: Comprehensive treatment programs (e.g., Early Start Denver Model, UCLA/Lovaas-based interventions), Focused interventions (e.g., social skill training), Treatments delivered by non-specialists (e.g., parent-mediated interventions), Technology-assisted interventions (e.g., video modeling), Medical treatments (e.g., risperidone), Complementary and Alternative Medicine (e.g., acupuncture), and Other (e.g., weighted vests). We selected these groups of treatments, as they might be most informative for LMIC. With respect to outcomes, we differentiated between communication and language skills, social behavior, joint attention, play, cognitive/intellectual functioning, academic performance/comprehension skills, restricted/repetitive/stereotyped behavior, challenging/problematic behaviors/irritability, hyperactivity, adaptive behavior, emotion regulation, ASD symptoms, daily living skills, sensory-motor skills, and others, respectively. Adverse effects were reported for medical as well as CAM treatments.

### Narrative summary of individual studies from LMIC

It turned out that none of the reviews addressed potential differences between HIC and LMIC even when studies from both classes of countries were considered. Therefore, we further analyzed the studies coming from LMIC, which were included in the reviews to find out which types of interventions are effective in LMIC and can be considered evidence-based. All empirical studies investigating outcomes of children and adolescents were analyzed in detail. Information on study design, number of participants, interventions investigated, and major findings were extracted. Findings were summarized in a qualitative way because research designs varied widely. In addition, the quality of the studies and evidence across studies was assessed. We used the What Works Clearinghouse standards (version 4.1, https://ies.ed.gov/ncee/wwc/Resources/ResourcesForReviewers) (76), as they are applicable to group-based and single-subject designs. Data extraction and rating of studies were performed by the first and last author independently. Initial agreement was 93 and 92%, respectively (see **Table 2**).

## Results

### Review selection

Electronic database search identified a total of 1,117 review papers. Fourteen additional reviews were found by manually searching reference lists. From a total of 1,131 review papers, 15 duplicate reviews were removed. One thousand one hundred sixteen review papers were assessed for eligibility, 973 of which were excluded based on abstract and title. The full text of the remaining 143 reviews was examined against the inclusion criteria. One hundred seven reviews were excluded because they failed to meet inclusion criteria, 46 reviews because they considered only studies from HIC. One review was not considered, because it could not be obtained (77). Finally, 35 reviews were included in the current meta-review (see Figure 1).

### Description of reviews

An overview of included reviews can be found in Table 1. Eight out of 35 reviews considered findings from HIC, UMIC, and LMIC, 21 from HIC and UMIC, three from HIC and LMIC, and three only from LMIC. Considering also the 46 reviews, which only included studies from HIC but met all other inclusion criteria (see Online Appendix for full list), this means that 32 of 81 reviews (40%) included research from HIC and other countries.

**Table 1:** Overview of systematic reviews including studies from high-income countries (HIC), lower middle-income countries (LMIC), and upper middle-income countries (UMIC).

**References**	**Year**	**Abbre- viated title**	**Age group**				**Countries of studies**			**Studies**			**Synthesis method**		**Systematic assessment of quality and/or evidence**	
			**Under 3 years**	**Pre- school (up to 6/7)**	**Up to 12**	**Adoles- cents 13-18**	**HIC**	**UMIC**	**LMIC**	**Total number**	**Single case studies**	**RCT**	**Quali- tative**	**Quanti- tative**	**Quality or evidence of individual studies**	**Evidence across studies**
Ameis et al. (78)	2018	Management of core and psychiatric symptoms	✓	✓	✓	✓	✓	✓ (Brazil, Iran)	✓ (India, Pakistan)	44	0	40	✓	✓	Cochrane's risk of bias tool (79)	Review specific
Bond et al. (80)	2016	Educational interventions	✓	✓	✓	✓	✓	✓ (South Africa)		85	54	30	✓		(81, 82)	Review specific [based on Wong et al. (47)]
Cheuk et al. (83)	2011	Acupuncture	✓	✓	✓	✓	✓	✓ (China)	✓ (Egypt)	10	0	10		✓	Cochrane's risk of bias tool (79)	No
Dababnah et al. (67)	2018	Autism interventions in India	✓	✓	✓	✓			✓ (India)	13	3	1	✓		No	No
Dawson-Squibb et al. (84)	2020	Parent education and training	✓	✓	✓	✓	✓	✓ (China, Turkey, Iran, Jordan)	✓ (India, Bangladesh, Tanzania)	37	0	5	✓		Mixed methods appraisal tool (85)	No
Dean and Chang (86)	2021	School-based social skills interventions	✓	✓	✓	✓	✓	✓ (China)		18	7	10	✓		(81, 87, 88)	No
Deb et al. (89)	2020	Parent training for Children with ASD	✓	✓	✓		✓	✓(Thailand)		17	0	15	✓	✓	Cochrane's risk of bias tool (90)	No
Dijkstra-de Neijs et al. (91)	2021	Play-based interventions	✓	✓	✓		✓	✓ (Iran)		32	0	32	✓	✓	Cochrane's risk of bias tool (92)	No
Ferguson et al. (93)	2019	Telehealth with behavior analytic interventions	✓	✓	✓	✓	✓		✓(Georgia)	28	18	4	✓		(81)	(81, 82)
Geretsegger et al. (94)	2016	Music therapy	✓	✓	✓		✓	✓ (Brazil)		10	0	10		✓	Cochrane's risk of bias tool (79)	GRADE system (95)
Harrop (96)	2015	Parent-mediated interventions	✓	✓			✓	✓ (Thailand)		29	2	19	✓		(81)	No
Koly et al. (97)	2021	Parent-mediated intervention programs	✓	✓	✓	✓			✓ (South Asian countries)	9	0	5	✓		The Kmet appraisal checklist (98)	No
Lee et al. (99)	2016	Movement-based interventions	✓	✓	✓	✓	✓	✓ (Iran)		14	9	5	✓		Mixed methods appraisal Tool (100)	No
Lee and Meadan (101)	2020	Parent-mediated interventions	✓	✓	✓	✓	✓	✓ (Albania, Brazil, China, Jordan, Macedonia)	✓ (India, Pakistan, Nigeria, Tanzania)	12	1	1	✓		No	No
Liu et al. (69)	2020	Parent-mediated interventions	✓	✓	✓	✓	✓	✓ (China)		21	0	16	✓	✓	Cochrane's risk of bias tool (90). QualSyst (98)	GRADE system (102)
Logan et al. (103)	2016	Augmentative and alternative communication interventions to increase communication		✓	✓	✓	✓	✓ (South Africa)		30	24	0	✓		Review specific [based on (104, 105)]	No
Mazon et al. (106)	2019	Technology-based interventions	✓	✓	✓	✓	✓	✓ (Brazil, Thailand, Romania)		31	0	13	✓	✓	(107)	No
McPheeters et al. (108)	2011	Medical treatments	✓	✓	✓		✓	✓ (Iran)		18	0	10	✓		Review specific	AHQR standards (109)
Mercer (110)	2017	DIR/Floortime™	✓	✓	✓		✓	✓ (Thailand)	✓ (India)	10	1	5	✓		No	No
Naveed et al. (111)	2019	Non-specialist mediated interventions	✓	✓	✓	✓	✓		✓ (India, Pakistan)	24	0	24		✓	Cochrane's Risk of Bias Tool (79)	GRADE system (112)
			**Under 3 years**	**Pre-** **school** **(up to** **6/7)**	**Up to 12**	**Adoles-** **cents 13-18**	**HIC**	**UMIC**	**LMIC**	**Total number**	**Single case studies**	**RCT**	**Quali-** **tative**	**Quanti-** **tative**	**Quality or evidence of individual studies**	**Evidence across studies**
Ona et al. (113)	2020	Pivotal response treatment	✓	✓	✓	✓	✓	✓ (Iran)		7	0	7	✓	✓	Cochrane's risk of bias tool (114)	GRADE system, (115, 116)
Oono et al. (117)	2013	Parent-mediated early interventions	✓	✓			✓	✓ (Thailand)		17	0	17		✓	Cochrane's risk of bias tool (79)	GRADE system (118)
Patra and Kar (119)	2020	Autism spectrum disorder in India	✓	✓	✓	✓			✓ (India)	26	4	3	✓		No	No
Pi et al. (120)	2021	Technology-assisted parent-mediated interventions	✓	✓	✓		✓	✓ (Macedonia)		16	0	16	✓	✓	(81, 82)	GRADE system
Piwowarczyk et al. (121)	2017	Gluten- and casein-free diet	✓	✓	✓	✓	✓		✓(Indonesia)	6	0	6		✓	Cochrane's risk of bias tool (122)	No
Sathe et al. (123)	2017	Nutritional and dietary interventions	✓	✓	✓		✓	✓ (Iran)	✓ (Egypt, Indonesia)	22	0	19	✓		(124)	Review specific (based on AHQR, 2014)
Siegel and Beaulieu (125)	2012	Psychotropic medications	✓	✓	✓	✓	✓	✓ (Iran)		33	0	33	✓		(81)	(81)
Smith and Iadarola (126)	2015	Psychological and behavioral interventions	✓	✓			✓	✓ (Thailand)		29	0	23	✓	✓	JCCAP criteria (127)	JCCAP criteria (127)
Spector (128)	2011	Sight word instruction		✓	✓	✓	✓	✓ (Turkey)		9	9	0	✓		(81)	(81)
Sullivan and Wang (129)	2020	Autism spectrum disorder interventions	✓	✓	✓	✓		✓ (China)		33	14	9	✓		No	No
Syriopoulou-Delli and Gkiolnta (130)	2020	Assistive technology		✓	✓	✓	✓	✓ (Malaysia, Romania)		13	1	1	✓		No	No
Tan et al. (131)	2021	Probiotics, prebiotics, synbiotics, and fecal microbiota transplantation		✓	✓	✓	✓	✓ (China, Thailand)	✓ (Egypt)	13	0	7	✓		Cochrane's risk of bias tool (132)	No
Tseng et al. (133)	2020	Social cognitive interventions				✓	✓	✓ (China)	✓ (Kenya)	18	0	18	✓		No	No
Vetter (134)	2018	Parent-child interaction therapy	✓	✓	✓		✓	✓ (Iran)		9	2	0	✓		No	No
Weitlauf et al. (124)	2017	Interventions targeting sensory challenges	✓	✓	✓		✓	✓ (Brazil, Iran, Thailand Turkey)		24	0	20	✓		Review specific	Review specific [based on (135)]

Reviews included between 6 and 85 different studies (M = 21.3, SD = 14.1). Reviews included either none or only very few studies from LMIC. Across all reviews, only 29 studies from LMIC investigating children's and adolescents' outcomes could be identified (see Table 2).

**Table 2:** Studies from LMIC included in systematic reviews from 2011 to 2021 investigating the effectiveness of treatments.

**Type of treatment**	**References**	**Year**	**Age group (Years)**	**Country**	**Study design**	***N***	**Inter- vention**	**Control if applicable**	**Primary outcome**	**Major findings**		**Group-designs**						**Single case designs**						**Overall quality rating**
											**Quality assessment**	**Randomized assignment**	**Acceptable attrition**	**Baseline equilvalence**	**Eligible outcome**	**No confounding**	**Data available**	**Experimental manipulation** **independent variable**	**Interassessor agreement**	**No residual treatment effects**	**Enough data points**	**Eligible outcome**	**No confounding**	
Comprehensive treatment programs	Gupta (136)	2015	5	India	Single case	1	Program based on theory of mind		Social behavior, ASD symptoms	Improved theory of mind and ASD symptoms							Yes	No	No	NA	Yes	Yes	Yes	Does not meet standards
Comprehensive treatment programs	Karanth et al. (137)	2010	2–6	India	Uncontrolled group design (pre vs. post)	30	Program based on ABA: “Communication DEALL”		Social-communication skills, adaptive skills, problematic behavior	Significantly improved communication skills, undesirable behavior reduced post intervention		No	Yes	NA	Yes	No								Does not meet standards
Focused interventions	Banerjee and Ray (138)*	2013	4–14	India	Controlled group design	20	Play therapy plus other regular management program	Other regular management program	Communication, problematic behavior, cognition, social behavior	According to abstract: improvements in communication and social skills given play therapy														
Focused interventions	Lal (139)	2010	9–12	India	Uncontrolled group design (pre vs. post)	8	Alternative and augment. Communication program		Communication and language skills, social behavior	Significantly improved language and communication skills, improved social behavior		No	Yes	NA	Yes	No								Does not meet standards
Focused interventions	Lal and Chhabria (140)	2013	3–6	India	RCT	26	Floor time intervention based on DIR model	Usual early intervention	Social behavior	Significant improvement of the treatment group, treatment group superior to control group at post test		Yes	Yes	Yes	Yes	Yes								Meets standards without reservations
Focused interventions	Malhotra et al. (141)	2010	7	India	Single case	1	Picture exchange communication system (PECS)		Communication and language skills	Improvements in communication skills and repetitive behavior							No	No	No	NA	Un- clear	Yes	Un-clear	Does not meet standards
Focused interventions	Rai et al. (142)	2015	9	India	Single case	1	Social stories		Problematic behavior	Improvement of undesirable behavior							Yes	No	No	No	Yes	Yes	Yes	Does not meet standards
Non-specialist mediated interventions	Bello-Mojeed et al. (143)	2016	3–17	Nigeria	Uncontrolled group design (Pre vs. Post)	20	Parent mediated behavioral intervention		Problematic behavior	Significantly reduced aggressive and self-injurious behaviors		No	Yes	NA	Yes	No								Does not meet standards
Non-specialist mediated interventions	Divan et al. (144)	2019	2–7	India	RCT	40	Community health-workers mediated intervention	Usual care	Autism symptom severity, Dyadic social communication, Adaptive behavior	Significantly improved autism severity scores and dyadic social communication skills with large effect size		Yes	Yes	Yes	Yes	Yes								Meets standards without reservations
Non-specialist mediated interventions	Juneja et al. (145)	2012	1.5–6	India	Uncontrolled group design (Pre vs. Post)	16	Individualized parent mediated behavioral intervention		ASD symptoms	Significant improvements in ASD symptom severity, social and language skills		No	Un- clear	NA	Yes	No								Does not meet standards
Non-specialist mediated interventions	Krishnan et al. (146)	2016	4	India	Uncontrolled group design (Pre vs. Post)	77	Parent-mediated multi-component, early intervention		Sensory, motor, and adaptive skills	Significant improvement of developmental age, motor skills, and cognitive performance		No	Un- clear	NA	Yes	Un- clear								Does not meet standards
Non-specialist mediated interventions	Louis and Kumar (147)	2015	2.5–5	India	RCT	30	Home-based program with additional training for fathers	Home-based program	Language, adaptive skills, and problematic behaviors	Significant improvement in social-communication skills, adaptive behaviors, and repetitive behaviors		Yes	Un- clear	Yes	Yes	Yes								Meets standards with reservations
Non-specialist mediated interventions	Manohar et al. (148)	2019	2–6	India	RCT	50	Brief parent-mediated intervention	Treatment as usual	Joint attention, imitation, social and adaptive skills	Significant improvement in autism severity, joint attention, dyadic interaction, language and communication skills, adaptive and intellectual functions		Yes	Yes	Yes	Yes	Yes								Meets standards without reservations
Non-specialist mediated interventions	Nair et al. (149)	2014	2–6	India	Uncontrolled group design (Pre vs. Post)	52	Low-intensity, parent-mediated early intervention		ASD symptoms, social behavior, communication and language skills	Significant improvements in ASD symptom severity, social and language skills		No	Un- clear	NA	Yes	No								Does not meet standards
Non-specialist mediated interventions	Rahman et al. (71)	2016	2–9	India and Pakistan	RCT single blind	65	Parent-mediated intervention for ASD in South Asia (PASS) + treatment as usual	Treatment as usual	Parent child interaction	Significantly better parent-child interaction after PASS with large effect size, no differences with respect to problematic behavior and communication skills		Yes	Yes	Yes	Yes	Yes								Meets standards without reservations
Technology assisted interventions	Barkaia et al. (150)	2017	4–6	Georgia	Single-case experiment design: multiple baseline	3	Telehealth coaching of therapists		Communi-cation and language skills	Some effects on language in all three children							Yes	Yes	Yes	NA	Yes	Yes	Yes	Meets standards without reservations
Technology assisted interventions	Lahiri et al. (151)	2015	13–18	India	Single case	8	Virtual reality technology (computer assisted)		Social-communication skills	Improvements in socio-communication skills and language in individuals with ASD							Yes	No	No	No	Yes	Yes	Yes	Does not meet standards
Technology assisted interventions	Lal and Bali (152)*	2007	5–10	India	Controlled trial	30	Visual strategy training	unclear	Communication and language skills	According to abstract: improvements in communication skills														
Technology assisted interventions	Padmanabha et al. (153)	2019	3–12	India	RCT	40	Home-based sensory interventions+ speech therapy and ABA	Speech therapy and ABA	Sensory skills	Significant reduction in sensory abnormalities and improvement in overall wellbeing and health-related quality of life		Yes	Yes	Yes	Yes	Yes								Meets standards without reservations
Technology assisted interventions	Paul et al. (154)	2015	3–4	India	Single case design: alternating treatment	3	Music-based intervention		Social-communication skills	Sung instructions were more effective for improving socio-communicative responsiveness than spoken language							Yes	Yes	Yes	NA	Yes	Yes	Yes	Meets standards without reservations
Medical treatments	Nagaraj et al. (155)	2006	2–9	India	RCT double blind	40	Risperidone	Placebo	Problematic behaviors	Significant reductions in problematic behaviors given risperidone, improvement in social- communication skills, weight gain, and increased sedation		Yes	Yes	Yes	Yes	Yes								Meets standards without reservations
Medical treatments	Desousa (156)	2010	5–16	India	Controlled group design	40	Risperidone	Fluoxetine	Problematic behaviors	Improvement in irritability and hyperactivity given risperidone, improvement of speech and stereotypy behavior given fluoxetine		No	Un- clear	Yes	Yes	No								Does not meet standards
Complemen -tary and alternative medicine	Allam et al. (157)	2008	4–7	Egypt	RCT single blind	20	Scalp acupuncture plus language therapy	Language therapy alone	Communication and language skills	Significant improvement in some aspects in both groups, more improvement in attention and receptive semantics given additional acupuncture		Yes	Yes	Yes	Yes	Yes								Meets standards without reservations
Complemen -tary and alternative medicine	Fahmy et al. (158)	2013	2.5–8.5	Egypt	RCT double blind	30	L-Carnitine	Placebo	ASD symptoms	Significantly stronger improvement given L-Carnitine therapy (but less severe symptoms in Placebo-group at baseline)		Yes	Yes	No	Yes	Yes								Meets standards without reservations
Complemen -tary and alternative medicine	Narasingharao et al. (159)	2017	5–16	India	Controlled trial	64	Yoga	School curriculum	Sleep, gastro -intestinal, behavior problems	Improvement in all three areas in yoga group but not in control group		No	Yes	Yes	Yes	Un- clear								Does not meet standards
Complemen -tary and alternative medicine	Pusponegoro et al. (160)	2015	4–7	Indonesia	RCT double blind	74	Diet with gluten and casein supplement	Diet without supplement	Problematic behaviors	Significant decrease of maladaptive behavior in both groups. No difference between groups.		Yes	Yes	Yes	Yes	Yes								Meets standards without reservations
Complemen -tary and alternative medicine	Radhakrishna (161)	2010	8–14	India	Single case	6	Yoga		Imitation skills	Improvement in children's non-verbal communication skills, cognitive skills, and social behavior							No	No	No	NA	Un- clear	Yes	Un- clear	Does not meet standards
Complemen -tary and alternative medicine	Saad et al. (162)	2015	3–9	Egypt	RCT double blind	101	Digestive enzymes	Placebo	ASD symptoms	Significantly better improvement in emotional response, ASD symptoms, and behavior given digestive enzyme treatment		Yes	Yes	Yes	Yes	Yes								Meets standards without reservations
Complemen -tary and alternative medicine	Shaaban et al. (163)	2018	5–9	Egypt	Uncontrolled group design (Pre *vs*. Post)	30	Probiotics		ASD symptoms and gastrointestinal symptoms	Significant improvements in the severity of ASD and gastrointestinal symptoms		No	No	NA	Yes	No								Does not meet standards

*Only abstract could be obtained.

Only randomized control trials (RCTs) were considered in 10 reviews, one considered only single case studies, the remaining included studies with various research designs. To integrate the findings, 22 (63%) provided only a qualitative synthesis, 5 (14%) only a quantitative synthesis, and 8 (23%) reported both. None of the reviews analyzed potential differences in the effectiveness of treatments for different classes of countries. This is also true for the eleven reviews, which included studies from HIC and LMIC.

Twenty-seven reviews (77%) assessed the quality or evidence provided by each individual study using a specific methodology. These methodologies varied considerably between studies. Most often Cochrane's risk of bias tool (79, 164) and the criteria proposed by Reichow et al. (81) were used. Fifteen (43%) assessed the evidence for types of treatments across studies using a specific methodology. Again, methodologies varied substantially. The Grades of Recommendation, Assessment, Development, and Evaluation (GRADE) system (95, 112) and the criteria by Reichow et al. (81) were most frequently utilized.

### Evidence for different types of treatment

Although most of the thirty-five reviews focused on a particular topic [e.g., parent-mediated early interventions, (117), see Table 1], many of the thirty-five reviews addressed more than one type of treatment according to our classification [e.g., comprehensive treatment programs, focused interventions, and parent-mediated interventions in the case of (117)]. Six reviews including 36 studies (two from LMIC) addressed comprehensive treatment programs; 14 reviews including 191 studies (five from LMIC) addressed various focused interventions; 15 reviews including 203 studies (eight from LMIC) addressed non-specialist mediated interventions; 15 reviews including 171 studies (five from LMIC) technology-assisted interventions, four reviews including 65 studies (two from LMIC) medical interventions, and 11 reviews including 67 studies (seven from LMIC) complementary and alternative medicine interventions. Hence the percentage of studies from LMIC ranged from 2.6% for focused interventions to 10% for complementary and alternative medicine. In the individual reviews only very few studies came from LMIC (0–4). This was true even for the three reviews that focused on research from LMIC (67, 97, 119). Many of the cited studies in these reviews did not investigate the ASD-related outcomes for children or adolescents.

The following sections first briefly describe which types of treatments are judged evidence-based in systematic reviews which only consider research from HIC (see Online Appendix for more detailed information on these reviews and their findings). Second, the results of the individual studies from LMIC for the respective type of treatment are summarized and their quality as well as the quality of the evidence is evaluated. Details on the studies can be found in Table 2.

#### Comprehensive treatment programs

As described above, comprehensive treatment programs integrate various types of interventions (i.e., applied behavior analysis, early intensive behavioral interventions, the UCLA young autism program by Lovaas and colleagues, ESDM, ToM, LEAP, TEACCH as well) over a prolonged period and are usually designed for preschoolers. The specific interventions differ between programs (60, 165).

There was consensus across reviews from HIC that comprehensive Applied Behavior Analysis (ABA) programs are evidence-based (126, 166, 167) as are Lovaas-based programs [UCLA Early Autism Project; (167, 168)].

With respect to LMIC, one single case study from India was cited in the reviews (136). It reported a positive effect of a comprehensive program on ASD symptoms and theory of mind. Another study investigated DEALL (Developmental Eclectic Approach to Language Learning), an indigenous early intervention program for children with autism, using an uncontrolled pre-post group design (137). Significant improvements in social-communication skills, motor skills, adaptive behaviors, language, and reduced behavioral problems were found.

The quality of the studies was low. The single case study did not meet the standards due to the absence of an experimental manipulation of the independent variable (136). The other study (137) did not meet the standards for group designs due to missing controls (see Table 2).

#### Focused interventions

Overall, focused interventions addressing social behavior (e.g., social skill training, play-based interventions, social stories) were considered established evidence-based treatments [(80, 91, 111, 169, 170); see also (171–173)]. The same was true for educational interventions aiming to improve academic performance by discrete skills teaching, response prompting strategies, and self-determination instructions (80, 169, 174). In addition, reviews judged joint attention-based interventions as evidence-based (80, 111, 167, 175).

Regarding LMIC, two reviews (67, 119) summarized studies from India on various focused interventions. One uncontrolled study investigated a vocabulary language program and reported significant improvement in language and social-communication skills at posttest (139). One single case study reported a positive effect of PECS on communication and repetitive behaviors while another single case study found that social stories reduced problematic behavior (141, 142). One RCT (140) found that a Developmental, Individual-Difference, Relationship-Based (DIR)/Floortime™ intervention improved social behavior. A second controlled study (138) reported a positive effect of play therapy in addition to the regular treatment (see Table 2 for details).

According to the WWC Procedures and Standards Handbook, one RCT met all basic design standards (140). The other controlled trial could not be evaluated because only the abstract could be obtained (138). The uncontrolled trial failed to meet standards (139) as did the two single case studies [(141, 142); see Table 2 for details].

#### Non-specialist mediated interventions

Most reviews focused on parent-mediated interventions, rather few considered peer-mediated interventions or teacher-mediated interventions (cf. Table 1). Several reviews judged parent training or parent-mediated interventions as evidence-based [(43, 117, 126, 167, 175, 176); see also (173)]. There was a consensus among two reviews on peer-mediated interventions (80, 111) that these interventions are effective and can be considered evidence-based. There is also some evidence for the effectiveness of teacher-implemented interventions (43, 126).

Regarding LMIC, four recent reviews looked into parent-mediated interventions for children with ASD (84, 97, 101, 119). However, most of the cited studies did not investigate the outcomes for children but looked into outcomes for parents (e.g., gain in knowledge, perceived helpfulness of intervention). Eight studies did assess ASD-related outcomes in children. Three uncontrolled studies from India (145, 146, 149) found that parent-mediated interventions improved ASD symptoms as well as social and language skills, sensory-motor and adaptive skills. One RCT from India and Pakistan (71) investigated the effectiveness of the Parent-mediated intervention for Autism Spectrum Disorder in South Asia (PASS), which is a program adapted from a program developed in the UK. They found that adding the program to a treatment as usual resulted in better parent-child interaction, but no further improvement in other outcomes. A second RCT from India by Manohar et al. (148) reported significant improvements in child-related measures such as autism severity, joint attention, social-communication skills, and adaptive behavior after a parent-mediated intervention. A third Indian RCT by Divan et al. (144) found positive effects of a community health worker-mediated communication intervention on autism severity scores and dyadic social communication skills. Louis and Kumar (147) found a positive effect of an intensive training for fathers added to a home-based program on social-communication skills, adaptive behaviors, and repetitive behaviors. One uncontrolled trial from Nigeria reported a significant effect of a parent-mediated behavioral intervention on challenging behaviors (143).

By WWC criteria, three RCTs were of good quality because they fulfilled the standards without reservations (71, 144, 148). One RCT (147) fulfilled the standards with reservations due to missing information on the randomization procedure and attrition. Four uncontrolled studies (pre-post design) did not meet the standards due to a lack of a control group (143, 145, 146, 149). According to the WWC standards at least two studies meeting standards without reservations are required for an intervention to be eligible for being considered evidence-based. There were three well-conducted RCTs. However, only one of the two studies investigating parent-mediated interventions (71, 148) reported positive effects on ASD symptoms. The third study (144) concerned community-health worker mediated interventions. In addition, <50% of the evidence comes from studies meeting standards without reservations. Hence there is not sufficient evidence.

#### Technology-assisted interventions

In line with previous research, we considered technology-assisted interventions to be interventions in which technology is the central feature supporting the acquisition of a goal of the learner such as social or academic skills, challenging behaviors, or daily living activities (44–47). Interventions include computer-based interventions, video-modeling music therapy, visual strategies training, video modeling, neurofeedback, Ayres Sensory Integration, and Augmented Auditory Integration. Overall, there was no consensus among reviews that technology-based interventions can be considered evidence-based, although promising results have been reported in quite a number of studies [see the reviews by (106, 120, 130, 177); for more information].

Regarding LMIC, one single case experimental study from Georgia investigating the effect of distance coaching of therapists found some effects of the intervention on the language skills of three children (150). A single case experimental study from India (154) found that sung instructions, as compared to spoken directives, were more effective in improving socio-communicative responsiveness in children. A non-experimental single case study from India on virtual reality-based interventions reported positive effects on social-communication skills [(151); see Table 2]. One controlled trial from India reported a significant effect of technology-based visual strategy training on communication skills (152). One RCT from India on home-based sensory interventions reported significant improvement in sensory abnormalities as well as overall wellbeing and health-related quality of life (153).

According to WWC Procedures and Standard Handbook, the two studies with single-case experimental designs met the standards (150, 154), while the other single case study did not (151). One RCT met all basic design standards (153). Unfortunately, the quality of one controlled trial could not be assessed as the full article could not be acquired (152).

#### Medical treatments

Overall, quite a number of reviews based on research from HIC considered aripiprazole and risperidone as evidence-based treatments for irritability, hyperactivity, repetitive behaviors, and inappropriate speech. However, significant side effects including marked weight gain and sedation were found for these medications (78, 108, 125, 178–180). Two Cochrane reviews pointed out lacking evidence for a long-term use of aripiprazole and risperidone (178, 180). The cited reviews also addressed other pharmacological treatments (including SSRIs, stimulants, sympatholytic agents, and chelating agents), none of which were considered evidence-based.

No evidence from LMIC was mentioned in the reviews until 2019. A more recent review (119), cites one RCT from India, which found risperidone to be effective in reducing behavioral problems (aggressiveness, hyperactivity, and irritability) and in improving social responsiveness and non-verbal communication skills (155). The same review also considers a non-randomized trial from India, which compared the efficacy and safety of risperidone and fluoxetine (156). A significant positive effect of risperidone on irritability and hyperactivity was found, while fluoxetine reduced speech deviance, social withdrawal, and stereotypic behavior. While the RCT met the standards of the WWC without reservations, the non-randomized trial did not. In addition, sample sizes were rather small.

#### Complementary and alternative medicine

In general, none of the treatments in this category have been considered evidence-based [see (78, 83, 111, 123, 124, 131)].

Regarding LMIC, one RCT from Indonesia found inconclusive results with respect to gluten and casein supplementation (160). One RCT from Egypt (162) investigated the effect of digestive enzymes and found significant improvement in emotional response and autistic behaviors. Another RCT from Egypt (158) showed an effect of L-Carnitine therapy in improving autistic behaviors. An RCT on acupuncture from Egypt (157) found that acupuncture in conjunction with language therapy may have an additional positive effect on some aspects of communication and language [see Cochrane review from (83), for more findings on acupuncture mostly coming from China a UMIC). A recent review from India (119) cited two studies on yoga. A controlled trial found that structured yoga improved gastrointestinal symptoms, sleep problems, and behavioral problems (159). A small uncontrolled study reported that integrated yoga therapy (IAYT) increased imitation skills (161). Another recent review by Tan et al. (131) cited one study from Egypt on probiotics and reported significant improvements in the severity of ASD and gastrointestinal symptoms (163). The four RCTs met the WWC standards without reservations, while the three other studies did not meet the standards (see Table 2 for more details).

#### Summary of evidence

Comprehensive treatment programs are well-investigated in HIC and some are considered evidence-based [see (47)]. Evidence from LMIC is lacking apart from two low-quality studies from India (136, 137), which entails that none of these programs can be considered evidence-based for LMIC.

Research on focused interventions also comes mostly from HIC [(61, 62); see (173), for a recent summary]. There are a few isolated studies on different types of focused interventions from LMIC, not providing sufficient evidence to consider them evidence-based. There were, however, two controlled studies looking into interventions addressing social-communication skills (a DIR/Floortime intervention and a play-based intervention) with reported positive findings (138, 140).

Non-specialist mediated interventions are particularly interesting for LMIC, as they require less resources and may be used to provide care for a larger number of children and adolescents. Reviews judged parent training or parent-mediated interventions as effective with good evidence, especially for preschoolers in HIC [(42, 43, 176); see also (173)]. In LMIC, however, the evidence is still insufficient to judged parent-mediated interventions as evidence-based. Two reviews from India and one review from Bangladesh judged parent-mediated interventions as effective (67, 97, 119). It is important to note, however, that in the review by Dababnah et al. (67) the total number of studies with respect to ASD was low. Two recent reviews by Patra and Kar (119) and Koly et al. (97) reported only three RCTs with good quality, while the other studies were mostly low-quality. Three other reviews also looked into parent-mediated interventions and/or parent training citing studies from LMIC (69, 84, 101). However, most of these studies did not look into children's outcomes in LMIC.

Many technology-based interventions have been tried for children and adolescents in HIC. For most interventions, high-quality evidence is still lacking. Recently, Steinbrenner et al. (173) judged video modeling and technology-aided instruction and intervention as evidence-based. Concerning LMIC, there is a lack of studies exploring the effect of technology-based interventions.

With respect to medical interventions, most studies were conducted in HIC focusing on the effect of pharmacological agents on behavioral problems. A new review from India [LMIC, (119)] included two studies on risperidone and found this medical agent to be effective for reducing behavioral problems (155, 156). These findings on antipsychotic medication conform to the findings in HIC.

Research on complementary and alternative medical treatments comes from HIC, UMIC, and LMIC. The existing evidence base is still too limited for the various types of CAM treatments. Again, evidence from LMIC is scarce and scattered across different treatments. Hence, none can be considered evidence-based.

## Discussion

In line with the significant increase in the prevalence of ASD in children and adolescents over the past two decades worldwide, a lot of research on different types of treatments for many different types of outcomes has been completed. Many systematic reviews have been published summarizing the respective research and more are published every year. We conducted a meta-review analyzing systematic reviews on the effectiveness of treatments and interventions in children and adolescents with ASD from 2011 until the end of 2021, which also considered research not coming from HIC. Our aims were to find out whether there are differences in the effectiveness of treatments in HIC *vs*. LMIC and which types of treatments can be considered evidence-based in LMIC.

### Summary of key findings

In this systematic review of reviews, we identified 35 systematic reviews that included research from LMIC and/or UMIC. Thirty-one of these considered also research from HIC. In the same time span (2011–2021) another 46 reviews on interventions for children and adolescents with ASD were published only including studies from HIC. There are many potential reasons why research studies from LMIC (and UMIC) may not be included in a systematic review. One is that these studies may be difficult to find and/or obtain. Another is that many of the studies were not RCTs and many not of high quality. Thus, these studies may have been excluded due to the inclusion criteria of the respective review.

Although eleven of the identified reviews included research from HIC and LMIC, none of the reviews looked for potential differences in effectiveness for a particular type of intervention. One obvious reason was the low number of studies from LMIC, which precluded any meaningful statistical comparison. Another seems to be lack of awareness that there may be relevant differences.

When we went back to the original studies from LMIC, which were cited in the reviews, we found studies with many different research questions, various research designs, and often a rather low quality. Nevertheless, we analyzed these studies and provided a narrative synthesis. Because of their heterogeneity, it did not make sense to integrate their findings statistically and compare them to the average findings from HIC. Thus, we were unable to determine, whether there are differences in the effectiveness of treatments in HIC and LMIC.

Finally, we evaluated the studies from LMIC for quality and evidence. Due to the low number of high-quality studies, no type of treatment fulfilled the criteria for being evidence-based according to the What Works Clearinghouse standards (version 4.1, https://ies.ed.gov/ncee/wwc/Resources/ResourcesForReviewers). One reason, why research from LMIC is still scarce, is probably the limited amount of funding available [cf. (33, 36, 37)]. Another reason may be that the awareness of the importance of research and knowledge about respective research methodologies is still moderate in some LMICs. A final reason might be that international publication fees are often prohibitively expensive, despite the price reductions for researchers from LMIC. This may reduce the international visibility of existing research.

### Limitations

Because of the many choices that have to be made when conducting a review of reviews with many methodological differences, some limitations exist. First, we decided to include only reviews that were published in English. Despite English being the common language of science, some reviews especially from LMIC might have been published in other languages. Therefore, some reviews and the findings summarized in them may be missing.

Second, we decided to include reviews being published between 2011 and the end of 2021 that consider research from HIC and LMIC and/or UMIC by searching only three electronic databases (PsycINFO, PubMed, Cochrane Database of Systematic Reviews). An updated version of this meta-review in the future should use more databases including databases collating research papers published in the languages of LMIC.

The third important decision, which limits our findings, was to consider only reviews that systematically reviewed the literature on interventions. While assessing the full texts we found some interesting unsystematic reviews of research from LMIC [e.g., (181)]. Following our inclusion criteria, we excluded these reviews. If the results from these unsystematic collections of research studies had been included, more research from LMIC might have been taken in account.

Fourth, we used the classification scheme of high-income, upper middle-income, and lower middle-income countries provided by the World Bank, which is the commonly used standard (e.g., by the WHO). This classification scheme is based on average income. As it does not specifically consider the health care system, some researchers have criticized using this classification scheme for making comparisons between countries [e.g., (101)]. They suggest to compare low-resource settings in health care to high resource settings instead.

### Implications for practice and future research

Although we were able to identify 35 systematic reviews summarizing the results from many empirical studies, there was very little evidence from LMIC. The eleven reviews including research from LMIC ran no analyses comparing results from HIC and LMIC. This finding has two important implications. First, more research needs to be conducted in LMIC on the effectiveness of different treatments and interventions for children and adolescents with ASD. The research should be of high quality no matter whether single case experimental designs or randomized group-based designs are used. Second, findings for HIC and LMIC need to be compared systematically. HIC and LMIC countries differ in many respects, including differences in health care systems but also in cultural and medical traditions. Hence, findings from HIC on specific treatments cannot be easily transferred to LMIC. There is no alternative to conducting the respective studies and to comparing the findings.

Nevertheless, there are some tentative implications for practice in LMIC. Many of the treatments that have been established as evidence-based by previous research, have to be considered as evidence-based only for HIC (see https://mn.gov/mnddc/asd-employment/pdf/09-NSR-NAC.pdf and https://www.nationalautismcenter.org/national-standards-project/phase-2/ for a good overview of these treatments). As shown in the present meta-review, there is currently not sufficient evidence for these interventions and treatments in LMIC to consider them evidence-based. There seems to be one notable exception: parent-mediated interventions. The reviews by Dababnah et al. (67), Koly et al. (97), Lee and Meadan (101), and Patra and Kar (119) concluded that these interventions are effective in LMIC. The evidence, however, was mostly indirect showing that parents acquire more knowledge and skills through these interventions. As shown here, direct evidence with respect to children's outcomes is still limited and studies were often of low quality. The review by Reichow et al. (43), however, supports the conclusion of the four reviews by showing that educating parents to deliver behavioral interventions is effective to address developmental disorders in LMIC. Thus, parent-mediated interventions can be considered at least promising and probably effective.

Another interesting option for LMIC might be the delivery of interventions by paraprofessionals, e.g., nurses, teaching assistants, social workers [cf. (182)]. At present respective research is almost completely lacking, but it might be interesting to explore this option in the future [see (144) for a first trial]. It also important to note that other evidence-based treatments and interventions from HIC might be promising for LMIC when being adapted to the respective context. Given the biological basis of ASD and the similar presentation of ASD in LMIC and HIC, treatments could work in both contexts.

## Conclusion

Treatments for children and adolescents with ASD, which are considered evidence-based in HIC, are still rarely investigated in LMIC. The findings presented here may still support mental health researchers, government organizations, and NGOs that seek to improve an uptake of effective treatments for children with autism in LMIC by summarizing the present state of research and pointing out, what evidence is still missing. It also shows that parent-mediated interventions at present have the best evidence for being effective, although the evidence is not sufficient when high standards are applied. We hope that the overview of reviews considering studies from LMIC and/or UMIC provides an easy access to mental health professionals (both specialists and non-specialists) in LMIC to the respective research. We recommended mental healthcare providers, clinicians, and other caregivers to look into these reviews and maybe even individual studies for more details on the specific treatments and interventions. This information along with their personal experience may allow them to engage in evidence-based practice when delivering treatments to children and adolescents with ASD.

## Data availability statement

The original contributions presented in the study are included in the article/Supplementary material, further inquiries can be directed to the corresponding author.

## Author contributions

MP, HA, and YH contributed to the conception and design of the meta-review. MP organized the database, wrote the first draft of the manuscript, and extracted the data. MP and YH made the bibliographic search and selected papers for the meta-review. HA contributed with comments to the draft, especially the introduction and the LMIC context. All authors contributed to the revision of the first draft, read, and approved the submitted version of the manuscript.

## Funding

This work was supported by a grant from the University of Goettingen, Germany and Ministry of Science and Technology, Government of Bangladesh.

## Conflict of interest

The authors declare that the research was conducted in the absence of any commercial or financial relationships that could be construed as a potential conflict of interest.

## Publisher's note

All claims expressed in this article are solely those of the authors and do not necessarily represent those of their affiliated organizations, or those of the publisher, the editors and the reviewers. Any product that may be evaluated in this article, or claim that may be made by its manufacturer, is not guaranteed or endorsed by the publisher.
