# Supplementary material for: Effectiveness of interventions for children and adolescents with autism spectrum disorder in high-income vs. lower middle-income countries: An overview of systematic reviews and research papers from LMIC
Source: Front Psychiatry. 2022 Aug 4;13:834783. doi: 10.3389/fpsyt.2022.834783 (PMC9386527; doi:10.3389/fpsyt.2022.834783)
Supplement: Supplementary file 1 [file Table_1.docx]

| **Online Appendix: Overview of Systematic Reviews 2011-2021 on Interventions for Children and Adolescents diagnosed with ASD which consider only Research from high-income countries (HIC)** | | | | | | | | | | | | | | | | | | |  |
| --- | --- | --- | --- | --- | --- | --- | --- | --- | --- | --- | --- | --- | --- | --- | --- | --- | --- | --- | --- |
| Name | Year | | Abbreviated Title | | Age group | | | | | |  | | Studies |  | Synthesis method | | Systematic assessment of quality and/or evidence | | |
|  |  |  |  |  | Under  3 years | | Preschool (up to 6/7) | Up to 12 | | Adolescents  13-18 | Total  number | | Single case studies | RCT | Qualitative | Quantitative | Quality or evidence of individual studies | Evidence across studies | |
| Aldabas | 2020 | | Peer-mediated interventions (PMIs) | |  | | 🗸 | 🗸 | | 🗸 | 16 | | 16 | 0 | 🗸 |  | No | No | |
| Baril | 2017 | | Early Start Denver Model | | 🗸 | | 🗸 |  | |  | 10 | | 3 | 2 | 🗸 |  | Yes (Reichow et al., 2008, Reichow, 2011) | Yes (Reichow et al., 2008, Reichow, 2011) | |
| Boudreau | 2015 | | Peer-mediated pivotal response treatment | |  | | 🗸 | 🗸 | |  | 5 | | 5 | 0 | 🗸 |  | Yes (Reichow et al., 2008) | Yes (Reichow et al., 2008) | |
| Bozkurt | 2014 | | Social stories in teaching for social skills | |  | | 🗸 | 🗸 | | 🗸 | 32 | | 32 | 0 | 🗸 | 🗸 | Yes, review specific | No | |
| Brignell | 2018 | | [Communication interventions for minimally verbal children](https://www.cochranelibrary.com/cdsr/doi/10.1002/14651858.CD012324.pub2/full?highlightAbstract=effectiv%7Ctreatment%7Cfor%7Cautism%7Ceffective%7Cwith%7Cdisorders%7Cdisord%7Cchildren%7Cspectrum%7Creview%7Cfour%7Cof%7Ceffect%7Cchild) | | 🗸 | | 🗸 | 🗸 | |  | 2 | | 0 | 2 | 🗸 |  | Yes (Cochrane´s risk of bias tool, Higgins et al., 2017) | Yes (GRADE system, Guyatt et al., 2008) | |
| Cappadocia | 2011 | | Social skills group training | |  | | 🗸 | 🗸 | | 🗸 | 10 | | 0 | 1 | 🗸 |  | No | No | |
| Ching | 2012 | | Aripiprazole | |  | | 🗸 | 🗸 | | 🗸 | 2 | | 0 | 2 |  | 🗸 | Yes (Cochrane's risk of bias tool, Higgins, 2008) | Yes (GRADE system, Guyatt et al. 2011) | |
| Dubin | 2020 | | Naturalistic Interventions | | 🗸 | | 🗸 | 🗸 | |  | 25 | | 11 | 13 | 🗸 |  | No | No | |
| Fuller | 2020 | | Early Intervention | | 🗸 | | 🗸 |  | |  | 29 | | 0 | 29 |  | 🗸 | Yes (Cochrane´s risk of bias tool, Higgins et al., 2011) | No | |
| Fung | 2016 | | Pharmacologic Treatment | | 🗸 | | 🗸 | 🗸 | | 🗸 | 11 | | 0 | 11 |  | 🗸 | Yes (Cochrane's risk of bias tool, Higgins and Thompson, 2003) | Yes (GRADE system, Guyatt et al., 2008) | |
| Hillman | | 2018 | Child-centered play therapy |  | | 🗸 | | 🗸 |  | | 4 | 2 | | 0 | 🗸 |  | No | No | |
| Hirsch | | 2016 | Aripiprazole |  | | 🗸 | | 🗸 | 🗸 | | 3 | 0 | | 3 |  | 🗸 | Yes (Cochrane's risk of bias tool, Higgins and Green, 2011) | Yes (GRADE system, Guyatt et al. 2011) | |
| Hong | | 2016 | Primary caregiver-implemented communication intervention | 🗸 | | 🗸 | |  |  | | 11 | 11 | | 0 | 🗸 |  | Yes (Kratochwill et al., 2010; Maggin et al 2013) | No | |
| James | | 2016 | Chelation |  | | 🗸 | | 🗸 |  | | 1 | 0 | | 1 | 🗸 |  | Yes (Cochrane 's risk of bias tool, Higgins and Green, 2011) | No | |
| Kent | | 2020 | Play-based interventions | 🗸 | | 🗸 | | 🗸 |  | | 19 | 0 | | 19 | 🗸 | 🗸 | Yes (QualSyst, Kmet et al., 2004) | No | |
| Knight | | 2015 | Comprehension strategies for students |  | | 🗸 | | 🗸 | 🗸 | | 23 | 23 | | 0 | 🗸 |  | Yes (Reichow, 2011) | Yes (Reichow et al., 2008, Reichow, 2011) | |
| Knight | | 2013 | Technology-based interventions for academic skills |  | | 🗸 | | 🗸 | 🗸 | | 29 | 17 | | 0 | 🗸 |  | Yes (Honer et al., 2005, Gersten et al., 2005 NSTTAC, 2010) | Yes (Honer et al., 2005, Gersten et al., 2005) | |
| Kreslins | | 2015 | Psychosocial interventions for anxiety | 🗸 | | 🗸 | | 🗸 | 🗸 | | 10 | 0 | | 10 | 🗸 | 🗸 | Yes (Cochrane 's risk of bias tool, Higgins and Green, 2011 | No | |
| Krishnaswami | | 2011 | Secretin | 🗸 | | 🗸 | | 🗸 |  | | 8 | 0 | | 7 | 🗸 |  | Yes (Owens et al., 2010) | Yes (Owens et al., 2010) | |
| Kuhaneck | | 2020 | Occupational therapy | 🗸 | | 🗸 | | 🗸 | 🗸 | | 20 | 3 | | 3 | 🗸 |  | Yes (AOTA guidelines  for systematic reviews, AOTA, 2017) | Yes (AOTA guidelines  for systematic reviews, AOTA, 2017) | |
| Lorah | | 2021 | Mobile technology |  | | 🗸 | | 🗸 | 🗸 | | 9 | 9 | | 0 | 🗸 |  | Yes (Horner et al., 2005) | No | |
| Maw | | 2018 | Cognitive, developmental, and behavioral interventions for preschool-aged children | 🗸 | | 🗸 | |  |  | | 14 | 0 | | 14 | 🗸 | 🗸 | Yes (Cochrane’s risk of bias tool, Higgins, 2008) | Yes (Reichow, 2011) | |
| Moon | | 2020 | Mobile device applications |  | | 🗸 | | 🗸 |  | | 7 | 0 | | 7 | 🗸 | 🗸 | Yes (Cochrane risk of bias (RoB)-2 tool) | No | |
| Morin | | 2018 | Augmentative and alternative communication interventions | 🗸 | | 🗸 | | 🗸 | 🗸 | | 24 | 24 | | 0 | 🗸 |  | Yes (WWC Procedures and Standards Handbook, USDE, 2016) | Yes (WWC Procedures and Standards Handbook, USDE, 2016) | |
| Mrachko | | 2017 | Interventions by non-experts - social communication | 🗸 | | 🗸 | | 🗸 | 🗸 | | 7 | 7 | | 0 | 🗸 |  | Yes (Horner et al., 2005) | No | |
| Munsell | | 2018 | Interventions supporting self-management of life tasks |  | |  | | 🗸 | 🗸 | | 14 | 0 | | 5 | 🗸 | 🗸 | Yes (NTACT evidence hierarchy, no reference) | Yes (NTACT evidence hierarchy, no reference) | |
| Nahmias | | 2019 | Community‐based early interventions | 🗸 | | 🗸 | |  |  | | 33 | 0 | | 0 |  | 🗸 | No | No | |
| Parsons | | 2017 | Pragmatic language interventions | 🗸 | | 🗸 | | 🗸 | 🗸 | | 21 | 0 | | 21 | 🗸 | 🗸 | Yes (Kmet et al., 2004) | No | |
| Parsons | | 2017 | Parent-mediated Intervention training | 🗸 | | 🗸 | | 🗸 | 🗸 | | 9 | 2 | | 3 |  | 🗸 | Yes (Kmet et al., 2004) | No | |
| Qi | | 2018 | Social stories interventions |  | | 🗸 | | 🗸 | 🗸 | | 22 | 22 | | 0 |  | 🗸 | Yes (Kratochwill et al., 2010; Maggin et al 2013) | Yes (Kratochwill et al., 2010, 2013; Maggin et al., 2013) | |
| Ramdoss | | 2011 | Computer-based interventions |  | | 🗸 | | 🗸 | 🗸 | | 10 | 1 | | 0 | 🗸 |  | Yes (Schlosser and Sigafoos, 2007) | No | |
| Ratliff-Black | | 2021 | Parent-mediated interventions | 🗸 | | 🗸 | | 🗸 | 🗸 | | 18 | 13 | | 0 | 🗸 | 🗸 | No | No | |
| Reichow | | 2018 | Early intensive behavioral intervention (EIBI) | 🗸 | | 🗸 | |  |  | | 5 | 0 | | 1 |  | 🗸 | Yes (Cochrane's risk of bias tool, Higgins, 2008) | Yes (GRADE system, Guyatt et al., 2008) | |
| Reichow | | 2013 | Non-specialist psychosocial interventions given intellectual disability or lower-functioning ASD | 🗸 | | 🗸 | | 🗸 | 🗸 | | 29 | 0 | | 15 | 🗸 | 🗸 | Yes (Cochrane's risk of bias tool, Higgins, 2008) | Yes (GRADE system, no specific reference) | |
| Schoen | | 2019 | Ayres sensory integration intervention |  | | 🗸 | | 🗸 |  | | 3 | 0 | | 2 | 🗸 |  | Yes (CEC standards, Cook et al., 2015) | Yes (CEC standards, Cook et al., 2015) | |
| Shalev | | 2020 | Parent-Mediated Interventions | 🗸 | | 🗸 | | 🗸 | 🗸 | | 11 | 0 | | 8 | 🗸 |  | No | No | |
| Stray | | 2012 | Social stories |  | | 🗸 | | 🗸 | 🗸 | | 19 | 18 | | 1 | 🗸 |  | No | No | |
| Sturman | | 2017 | [Methylphenidate](https://www.cochranelibrary.com/cdsr/doi/10.1002/14651858.CD011144.pub2/full?highlightAbstract=effectiv%7Ctreatment%7Cfor%7Cautism%7Ceffective%7Cwith%7Cdisorders%7Cdisord%7Cspectrum%7Cchildren%7Creview%7Cfour%7Cof%7Ceffect%7Cchild) |  | | 🗸 | | 🗸 | 🗸 | | 4 | 0 | | 4 | 🗸 | 🗸 | Yes (Cochrane’s risk of bias tool, Higgins et al., 2017) | Yes (GRADE system, Grade working group, 2004, GRADEpro sofware (2015) | |
| Tomeny | | 2020 | Caregiver-Implemented Intervention | 🗸 | |  | |  |  | | 26 | 9 | | 13 | 🗸 |  | No | No | |
| Tachibana | | 2017 | Comprehensive interventions for pre-school children | 🗸 | | 🗸 | |  |  | | 33 | 0 | | 33 |  | 🗸 | Yes (Cochrane’s risk of bias tool, Higgins and Green, 2011) | Yes (GRADE system, Guyatt et al., 2008) | |
| Taylor | | 2017 | Weighted vests |  | | 🗸 | | 🗸 |  | | 7 | 7 | | 0 | 🗸 |  | Yes (Kratochwill et al., 2010, 2013) | Yes (Kratochwill et al., 2010, 2013) | |
| Vasa | | 2014 | Psychopharmacological and non-psychopharmacological treatments for anxiety |  | | 🗸 | | 🗸 | 🗸 | | 15 | 0 | | 8 | 🗸 |  | Yes (USPSTF, 2008) | Yes (GRADE system, Guyatt et al., 2008) | |
| Warren | | 2011 | Early intensive intervention | 🗸 | | 🗸 | | 🗸 |  | | 34 | 17 | | 2 | 🗸 |  | Yes, review specific (based on Berkman et al., 2013) | Yes (AHQR evidence grading, Berkman et al., 2013) | |
| Williams | | 2012 | [Secretin](https://www.cochranelibrary.com/cdsr/doi/10.1002/14651858.CD003495.pub3/full?highlightAbstract=effectiv%7Ctreatment%7Cfor%7Cautism%7Ceffective%7Cwith%7Cdisorders%7Cdisord%7Cchildren%7Cspectrum%7Creview%7Cfour%7Cof%7Ceffect%7Cchild) | 🗸 | | 🗸 | | 🗸 | 🗸 | | 16 | 0 | | 16 |  | 🗸 | Yes (Cochrane's risk pf bias tool, Higgins, 2008) | No | |
| Zarafshan | | 2016 | Non-pharmacological interventions for stereotyped and repetitive behaviors of pre-school children | 🗸 | | 🗸 | |  |  | | 15 | 12 | | 2 | 🗸 |  | No | No | |
| Zwaigenbaum | | 2015 | Early Interventions under 3 Years of Age | 🗸 | |  | |  |  | | 24 | 0 | | 12 | 🗸 |  | Yes (GRADE system, Guyatt et al., 2008) | Yes (GRADE system, Guyatt et al., 2008) | |
